# Clinical knee findings in floor layers with focus on meniscal status

**DOI:** 10.1186/1471-2474-9-144

**Published:** 2008-10-22

**Authors:** Søren Rytter, Lilli Kirkeskov Jensen, Jens Peter Bonde

**Affiliations:** 1 Department of Orthopaedics, Regional Hospital Viborg, Denmark; 2 Department of Occupational Medicine, Regional Hospital Skive, Denmark; 3 Department of Occupational Medicine, Aarhus University Hospital, Denmark

## Abstract

**Background:**

The aim of this study was to examine the prevalence of self-reported and clinical knee morbidity among floor layers compared to a group of graphic designers, with special attention to meniscal status.

**Methods:**

We obtained information about knee complaints by questionnaire and conducted a bilateral clinical and radiographic knee examination in 134 male floor layers and 120 male graphic designers. After the exclusion of subjects with reports of earlier knee injuries the odds ratio (OR) with 95% confidence intervals (CI) of knee complaints and clinical findings were computed among floor layers compared to graphic designers, using logistic regression. Estimates were adjusted for effects of body mass index, age and knee straining sports. Using radiographic evaluations, we conducted side-specific sensitivity analyses regarding clinical signs of meniscal lesions after the exclusion of participants with tibiofemoral (TF) osteoarthritis (OA).

**Results:**

Reports of knee pain (OR = 2.7, 95% CI = 1.5–4.6), pain during stair walking (OR = 2.2, 95% CI = 1.3–3.9) and symptoms of catching of the knee joint (OR = 2.9, 95% CI = 1.4–5.7) were more prevalent among floor layers compared to graphic designers. Additionally, significant more floor layers than graphic designers had clinical signs suggesting possible meniscal lesions: a positive McMurray test (OR = 2.4, 95% CI = 1.1–5.0) and TF joint line tenderness (OR = 5.4, 95% CI = 2.4–12.0). Excluding floor layers (n = 22) and graphic designers (n = 15) with radiographic TF OA did not alter this trend between the two study groups: a positive McMurray test (OR = 2.2, 95% CI = 1.0–4.9), TF joint line tenderness (OR = 5.0, 95% CI = 2.0–12.5).

**Conclusion:**

Results indicate that floor layers have a high prevalence of both self-reported and clinical knee morbidity. Clinical knee findings suggesting possible meniscal lesions were significant more prevalent among floor layers compared to a group of low-level exposed graphic designers and an association with occupational kneeling could be possible. However, causality cannot be confirmed due to the cross-sectional study design.

## Background

Workers in the construction industry are exposed to different kinds of knee strains. Floor layers are in particular exposed to repetitive and prolonged periods of kneeling work and only few jobs have the same level of knee demands as workers in this profession. External and internal knee stresses are high during kneeling work positions and may affect both intra- and peri-articular knee structures e.g. cartilage, menisci, cruciate and collateral ligaments, bursae and the patella tendon. Knee morbidity may be attributable to acute or chronic injuries on these structures and explain earlier reports of prolonged sick leave and premature retirement among floor layers. [1,2]

Several studies have focused on the association between kneeling and knee osteoarthritis (OA) and it has been shown that floor layers have an increased prevalence of knee OA. [3-9] However, reports of knee complaints among floor layers have been much higher than explainable by cases with knee OA alone. Knee morbidity may therefore be attributable to other pathologies than OA. In this respect there has been few reports concerning the association between occupational related factors and meniscal lesions. [10-13] Early studies in the 1950s and 1960s showed an increased prevalence of meniscal damage among miners who had a significant proportion of kneeling work in their work tasks and in a more recent case-control study of hospital treated meniscal injuries, Baker et al. showed that degenerative lesions were increased almost 4-fold among workers with occupational kneeling. [11-13] Few previous studies have focused on clinical knee morbidity among floor layers.[2,14-16] However, only one of these studies evaluated clinical signs of meniscal lesions and they found an even distribution of tibiofemoral (TF) joint line tenderness between floor layers and a reference group of painters.[14]

Due to sparse information in the literature concerning meniscal pathology among workers with kneeling work demands, the main objective of the current study was to evaluate the prevalence of clinical assessed meniscal lesions and self-reported knee complaints among floor layers compared to a group of low-level exposed graphic designers.

## Methods

### Study participants

A sample of male floor layers (n = 286) and male graphic designers (n = 370) were established in 1994 based on trade union rosters. Workers, who were members of the trade union for floor layers and the former graphical workers union 10 years earlier (1984), were also included. Members aged 36–70 years in 2004 and residents in Copenhagen (capital city) and Aarhus (second largest city), Denmark were included in the study. Graphic designers were included as reference group. They work at visual display units and their work do not include knee-demands. Danish floor layers and graphic designers are comparable regarding the level of education and socio-economic status.

**Figure 1:**
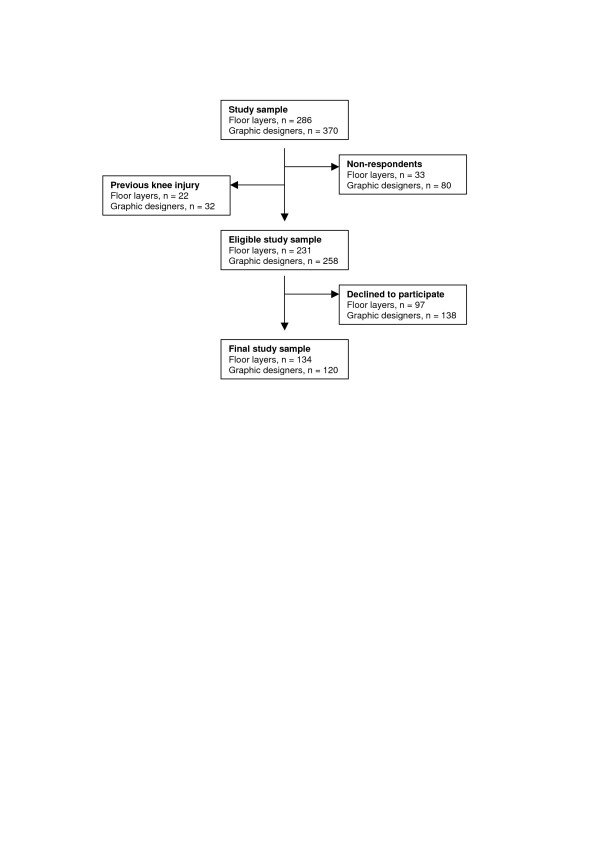
Flow chart on details of the study material.

A self-administered questionnaire was mailed to the initial study sample with a response rate of 88% and 78% among floor layers and graphic designers, respectively. Among respondents, subjects who reported a previous knee injury (fractures involving the knee joint, meniscal lesions or cruciate ligament ruptures) were excluded (floor layers, n = 22; graphic designers, n = 32). The remaining part of the cohort, 231 floor layers and 258 graphic designers, were offered a clinical and radiographic knee examination. Written informed consent was obtained from 134 floor layers and 120 graphic designers (figure 1). Participants in the final study sample (n = 254) also filled in a Knee Injury and Osteoarthritis Outcome Score (KOOS) questionnaire.[17]

Permission from the Central Region Committee on Biomedical Research Ethics in Denmark was obtained.

### Questionnaire data

The initial questionnaire mailed to the cohort addressed anthropometrical characteristics (age, height and weight), information about employment and labor marked connection (trade seniority), history of knee complaints (during the past 12 months) or knee injuries (fractures, menisci, cruciate ligaments or muscle injuries) and knee straining sports experience defined as ever participated in: football, handball, badminton, tennis, volleyball, basketball, ice hockey, weight lifting or skiing. Additional information about knee-specific complaints were obtained by the Danish version of the KOOS questionnaire among those who volunteered to participate in the medical examinations.[17] This questionnaire is a patient-administered questionnaire validated to assess information about knee complaints and associated problems, especially in the assessment of changes over time, induced by treatment due to earlier knee injuries or posttraumatic OA. However, reference values from subjects without signs of knee injuries have been established.[18] KOOS consists of 42 questions apportioned on 5 subscales of knee complaints and associated problems (during the last week). Each question has five standardized answer options scored from 0 to 4. In the current study we selected eight questions regarding knee pain/symptoms and physical function for the analyses (appendix) and each answer was dichotomized in accordance to the five answer options (0 vs. 1–4).

### Clinical knee examinations

Clinical examinations of both knees included inspection and palpation of the anterior and posterior knee region, assessment of joint mobility (range of motion), gross disturbance of patella tracking (patellar tracking test), clinical signs suggesting possible meniscal lesions (McMurray test and TF joint line palpation), registration of patellofemoral (PF) crepitation and knee effusion (patellar tap test). All diagnostic tests were performed with the participant lying supine, except the patellar tracking test and results was recorded as positive or negative findings. In the patellar tracking test the seated participant actively extend the knees from 90-degree flexion to full extension, registering any lateral displacement of the patella. A positive McMurray test was consistent with localized joint line pain or a palpable click bringing the knee from maximal flexion too extension, while the foot was retained in full external (medial meniscus) or internal (lateral meniscus) rotation.[19]

One examiner (SR), a postgraduate orthopedic fellow accustomed to clinical knee examinations, conducted all clinical examinations. To maximize consistency in each examination a standardized manual describing the pertinent physical tests was conducted and followed thoroughly. Participants were invited randomly and the examiner had no prior knowledge of which of the two occupations they were associated and furthermore unaware of any medical history of knee disorders.

### Radiographic knee examinations

Radiographs of both knees were obtained in the standing position with the knee in 20–30 degree flexion in all three views: postero-anterior (PA), lateral and axial of the PF joint space. A standardized examination technique with a device supporting the knee allowed adjustment without fluoroscopy for optimal visualization of the medial and lateral TF and PF joint spaces. [20,21]

Radiographs were read and scored on workstations with 2 K screens by one experienced musculoskeletal radiologist. Radiographic scoring comprised assessment of the medial and lateral joint spaces of both the TF and PF compartment using a modified Ahlbäck scale (grade 0–6) of joint space narrowing (JSN) and subchondral bone attrition.[22] The following grades were defined: grade 0 = normal; grade 1 = minimal but definite JSN (25% JSN); grade 2 = moderate JSN (50% JSN); grade 3 = severe JSN (75% JSN); grade 4 = obliteration of the joint space, "bone on bone but no attrition"; grade 5 = < 5 mm attrition of subchondral bone and grade 6 = ≥ 5 mm bone attrition. The main criteria for the assessment of minimal but definite JSN (grade 1) were comparisons between the same joint spaces of the right and the left knee in each participant and when both medial TF joint spaces were affected a minimal joint space of 4 mm was used.[23] According to this classification OA was defined as JSN ≥ 25% in at least one knee and patterns of involvement categorized into TF and PF OA.

### Analyses

The adjusted odds ratio (OR) with 95% confidence interval (CI) of knee complaints and clinical knee findings were computed on the subject level among floor layers compared to graphic designers. Using logistic regression, models were adjusted for age (≤ 49, 50–59, ≥ 60 years), body mass index (BMI; < 25, 25–29, ≥ 30 kg/m^2^) and knee straining sports activities (yes/no). Independent variables were kept in the models whether statistically significant or not.

In side-specific sensitivity analyses, clinical signs suggesting possible meniscal lesions were conducted after the exclusion of participants with radiographic TF OA. In addition the proportion of participants with subjective symptoms (knee pain during stair walking and/or catching of the knee joint) were computed among those with a positive clinical test (McMurray test or TF joint line tenderness) suggesting possible meniscal lesions.

The radiographic intra-reader reliability was tested in regard to the separation between a normal joint space and a minimal but definite JSN (25% JSN) as well as the scoring of different grades of JSN. All subjects scored with knee OA (n = 61) were randomly mixed by an IT-technologist in a file of digital images with the knees of 26 subjects scored as normal (n = 193). The same reader randomly and blindly rescored these radiographs (n = 87). The intra-reader agreement was 96.6% for assessment of the TF compartment and 96.5% of the PF compartment.

Statistical analyses were performed using Stata (version 8.0, StataCorp LP, College Station, TX).

## Results

### Individual characteristics

Characteristics of study participants are given in table 1. Mean age and trade seniority was higher among graphic designers than among floor layers. Participation rates varied between age groups and were higher among workers aged 50–59 years in both groups. Regarding height, weight and BMI the two occupational groups were comparable. The proportion of lifetime participation in any knee straining sports was higher among graphic designers (67%) compared to floor layers (53%).

**Table 1:** Characteristics of the final study sample, floor layers (n = 134) and graphic designers (n = 120)

**Characteristics**	**Floor layers**		**Graphic designers**	
Age (years) *mean, SD*	52.6	6.9	57.9	5.9
- ≤ 49 *n, %*	43	32.1	7	5.8
- 50–59 *n, %*	72	53.7	73	60.8
- ≥ 60 *n, %*	19	14.2	40	33.4
Seniority^† ^(years) *mean, SD*	29.2	10.2	35.6	8.6
Height (cm) *mean, SD*	179.1	6.6	177.5	6.3
Weight (kg) *mean, SD*	84.7	13.6	82.2	14.6
BMI^‡ ^(kg/m^2^) *mean, SD*	26.4	3.8	26.0	3.9

^† ^Duration of employment in the trade

^‡ ^BMI, body mass index

### Questionnaire reports

Questionnaire reports from the initial study sample among those who agreed to participate in the medical examinations and those who declined (figure 1) showed that subjects with knee complaints (during the past 12 months) were more willing to participate than subjects without. However, this selective participation was much more pronounced among graphic designers than among floor layers, especially in the younger and elderly age groups (table 2).

**Table 2:** Proportion of knee complaints among floor layers and graphic designers from the initial study sample, stratified into age groups

	**Floor layers**						**Graphic designers**					
	
	**Attendees^†^**		**Non-attendees^‡^**				**Attendees^†^**		**Non-attendees^‡^**			
	
**Age groups**	N^§^	n^||| ^(%)	N^§^	n^||| ^(%)	RR	95% CI	N^§^	n^||| ^(%)	N^§^	n^||| ^(%)	RR	95% CI
≤ 49 years	43	23 (53)	45	19 (42)	1.27	0.82–1.97	7	4 (57)	22	5 (23)	2.51	0.92–6.85
50–59 years	72	41 (57)	23	9 (39)	1.46	0.84–2.52	73	23 (32)	42	8 (19)	1.65	0.81–3.36
≥ 60 years	19	6 (32)	29	12 (41)	0.76	0.35–1.68	40	19 (48)	74	12 (16)	2.93	1.59–5.40

Risk ratio (RR) with 95% confidence interval (CI) among those who agreed to attend the medical examination compared to those who declined.

^† ^Floor layers (n = 134), graphic designers (n = 120)

^‡ ^Floor layers (n = 97), graphic designers (n = 138)

^§ ^Total in each age group

^|||^Knee complaints during the past 12 months

Floor layers participating in the medical examinations had a high prevalence of knee-specific complaints and associated problems corresponding to each of the directed questions (table 3). Compared to graphic designers, floor layers more often reported knee pain (monthly, weekly, daily or always), knee pain at night while in bed or pain during stair walking, sensation of knee joint stiffness after first wakening in the morning, catch or hang up of the knee joint when moving and swelling in the knees. Changing the dichotomisation of these answer options in the questionnaire from 0 vs. 1–4 to 0–1 vs. 2–4 did not alter the difference between the two occupational groups. Questions about knee difficulty during kneeling and squatting activities showed a smaller difference between the two study groups. However, restricting analyses to participants aged 50–59 years a significant difference between the two groups in relation to both squatting (OR = 2.8, 95% CI = 1.4–5.7) and kneeling (OR = 2.6, 95% CI = 1.3–5.4) was found. Non-significant results were found in the other two age strata.

**Table 3:** Self-reported knee complaints among floor layers (n = 134) compared to graphic designers (n = 120)

	**Floor layers**		**Graphic designers**			
	
**Questionnaire response**	n	%	n	%	OR _adj._^†^	95% CI
Knee pain^‡^	89	67.4	50	42.0	2.7	1.5–4.6
Knee pain at night^‡^	49	37.1	21	17.6	3.5	1.8–6.6
Knee pain during stair walking^‡^	86	65.2	52	43.7	2.2	1.3–3.9
Morning stiffness	78	58.2	48	40.0	1.9	1.1–3.2
Catch or hang up of the knee	42	31.3	16	13.3	2.9	1.4–5.7
Swelling of the knee	65	48.5	25	20.8	4.7	2.5–8.6
Difficult during squatting^§^	82	61.2	62	53.4	1.6	0.9–2.7
Difficult during kneeling^§^	91	67.9	63	54.3	1.8	1.0–3.1

Adjusted odds ratio (OR) with 95% confidence intervals (CI).

^† ^Adjusted for body mass index, age and knee straining sports

^‡ ^One graphic designer and two floor layers had not answered questions about knee pain

^§ ^Four graphic designers had not answered questions about difficult during squatting and kneeling

### Clinical findings

The most prevalent clinical finding among participants was PF crepitation, which was found among 65% of the floor layers and 55% of the graphic designers (table 4). However, a characteristic difference between the two study groups was the finding of a typical hyperkeratinisation of the prepatellar skin area (hyperkeratosis) among floor layers (OR = 14.0, 95% CI = 6.1–32.3). Clinical signs suggesting possible meniscal lesions e.g. tenderness on palpating the TF joint lines and a positive McMurray test was significantly more prevalent among floor layers compared to graphic designers and moreover evaluation of patellar stability by the patellar tracking test revealed significantly more observations of lateral patellar displacement among floor layers (OR = 8.3, 95% CI = 1.8–38.9). Apart from the proximal part of the patella tendon, tenderness on palpating different areas in the anterior aspect of the knee region did not show any disparities between the two study groups (table 4).

**Table 4:** Clinical findings according to worst affected knee among floor layers (n = 134) compared to graphic designers (n = 120)

	**Floor layers**		**Graphic designers**			
	
**Clinical knee findings**	n	%	n	%	OR_adj._^†^	95% CI
Prepatellar hyperkeratosis	62	46.3	8	6.7	14.0	6.1–32.3
Positive patellar tap test	8	6.0	9	7.5	0.8	0.3–2.4
Patellofemoral crepitation	87	64.9	66	55.0	1.9	1.1–3.4
						
Tenderness on palpating the						
- tibiofemoral joint line	42	31.3	11	9.2	5.4	2.4–12.0
- patella tendon proximally	7	5.2	1	0.8	9.4	1.1–82.3
- patella retinaculum	7	3.7	8	3.3	0.7	0.2–2.2
- pes anserinus	3	2.2	2	1.7	1.6	0.2–11.9
- tibial tuberosity	2	1.5	1	0.8	2.1	0.2–24.4
						
Positive McMurray test	32	23.9	14	11.7	2.4	1.1–5.0
Diminished flexion	4	3.0	1	0.8	4.1	0.4–41.2
Positive patellar tracking test	15	11.2	2	1.7	8.3	1.8–38.9

Adjusted odds ratio (OR) with 95% confidence interval (CI).

^† ^Adjusted for body mass index, age and knee straining sports

Analyses between floor layers and graphic designers without clinical findings of prepatellar hyperkeratosis revealed the same trend as those with such findings. Among the subgroup (floor layers, n = 72; graphic designers, n = 112) without hyperkeratosis, the adjusted OR for TF joint line tenderness was 4.7 (95% CI = 2.0–10.7) whereas results for a positive McMurray test was 3.3 (95% CI = 1.4–7.8) among floor layers compared to graphic designers.

### Radiographic findings

Twenty-four percent of the participants were classified as having radiographic knee OA. According to the worst affected knee and compartment there was a diverse distribution between the two occupations. Nineteen floor layers and nine graphic designers were classified as having isolated TF OA while isolated PF OA was found among nine floor layers and fifteen graphic designers, respectively. OA in both the TF and PF compartments was found in three floor layers and six graphic designers (table 5).

**Table 5:** Proportion of radiological determined knee osteoarthritis according to worst affected knee and compartment among floor layers (n = 134) and graphic designers (n = 120)

	**Floor layers**		**Graphic designers**	
	
**Knee osteoarthritis^‡^**	n	(%)	n	(%)
**Joint space narrowing ≥ 25%**				
- Tibiofemoral	10	(8)	2	(2)
- Patellofemoral	5	(4)	8	(7)
**Joint space narrowing 50–75%**				
- Tibiofemoral	7	(5)	7	(6)
- Patellofemoral	4	(3)	8	(8)
**Obliteration or bone attrition**				
- Tibiofemoral	5	(4)	6	(5)
- Patellofemoral	3	(2)	5	(4)

^‡ ^Combined TF and PF OA: floor layers, n = 3; graphic designers, n = 6

Regarding clinical signs suggesting possible meniscal lesions a significant difference between the two study groups remained after the exclusion of floor layers (n = 22) and graphic designers (n = 15) with radiographic TF OA: positive McMurray test (OR = 2.2, 95% CI = 1.0–4.9) and TF joint line tenderness (OR = 5.0, 95% CI = 2.0–12.5).

### Concomitant complaints and clinical findings

A high proportion of participants with positive clinical findings that could suggest possible meniscal lesions did also report complaints of knee pain during stair walking and/or catching of the knee joint. Analyses, including all participants (floor layers and graphic designers) in the final study sample, showed that the likelihood of reporting such complaints was 87,0% among those with a positive McMurray test and 86.8% among those with TF joint line tenderness. However, there were also a high proportion of participants with subjective complaints that did not have clinical signs of possible meniscal lesions. Among those with reports of knee pain during stair walking and/or catching of the knee joint, 68.5% did not have TF joint line tenderness and 72.6% had a negative McMurray test.

Knee pain during stair walking and/or catching of the knee joint was also a prevalent symptom (69.7%) among participants with PF OA and higher among floor layers (83.3%) compared to graphic designers (61.9%).

## Discussion

Assessed by reports of knee-specific complaints and clinical knee examinations results revealed a high prevalence of knee morbidity among floor layers and clinical signs suggesting possible meniscal lesions were especially prevalent compared to graphic designers. However, some limitations of the study warrant discussion. The most apparent is the effect of a primary and secondary healthy-worker selection that may be inventible in occupations with high physical demands. Such selection mechanisms would typically result in an underestimation of the investigated association. Taking this issue into account current results still revealed a high prevalence of both knee complaints and clinical signs of knee morbidity among floor layers. Another limitation may be due to selection bias. Although, a high questionnaire response rate among the initial study sample there was a high drop out rate among those offered a clinical and radiographic knee examination; 38% of the floor layers and 48% of graphic designers. Results could be biased if the decision to participate were differentially influenced by previous or current knee complaints. Analyses of questionnaire reports among those who participated in the medical examinations (n = 254) and those who declined (n = 235) revealed a higher participation rate among graphic designers with knee complaints than among floor layers. Part of the explanation could be that graphic designers with knee complaints may be more motivated to participate in a medical knee examination than graphic designers without complaints, whereas floor layers who are depended on well-functioning and healthy knees may participate having knee complaints or not. Furthermore, participation rates differed strongly between age groups and were particularly low among participants younger than 50 years and older than 60 years. It is therefore most likely that selective participation has influenced estimates towards the null, especially in the young and elderly age groups of the study sample. Anyhow, sensitivity analyses with restriction to the age stratum (50–59 years) with high participation rates in both floor layers and graphic designers corroborated main findings. The use of one examiner is also a limitation that potentially may implement bias, as the outcome of the examinations was reliant on one-examiner skills and knowledge. To minimize this impact all tests and clinical maneuvers were trained beforehand according to a standardized examination manual and the examiner was furthermore blinded to any medical history of knee disorders and occupational affiliation. Blinding the examiner in regard to occupational affiliation was difficult due to a diverse distribution of prepatellar hyperkeratosis among the two study groups. However, analyses among the subgroup without such skin changes did not alter results regarding the clinical judgment of possible meniscal lesions. Still, we have to acknowledge the possibility of differential misclassification due to chatting with study participants during examinations.

Knee symptoms and clinical signs of actual meniscal pathology is a complicated issue. It is only the outer one-third of the menisci that has a neural innervation.[24] Structural meniscal lesions do therefore not necessarily correlate with knee symptoms. Indeed, several studies have shown a high baseline prevalence of meniscal lesions in asymptomatic knees, especially among older adults. [25-28] Nevertheless, several clinical tests have been described for the primary assessment of possible meniscal lesions and TF joint line tenderness and the McMurray test are among the most commonly used.[29] Previous reports and reviews evaluating the accuracy of clinical tests have shown heterogeneous results and some studies state that these tests are of little value in the clinical practice. [30-35] However, decisive determinants that may influence the accuracy of such clinical tests could be due to a variability in study populations and differences in the performance and interpretation of clinical outcomes.[35] Differential recruitment of hospital-referred patients with suspected knee pathology may for example influence the accuracy in such studies. Moreover, the diagnostic performance and interpretation of tests may differ between studies. Performance of the McMurray's test may for instance be with or without valgus/varus stress and interpret as pain solely or with/without a click at the corresponding joint lines. In the current study we conducted clinical examinations among participants without presently acute knee traumas and performed the McMurray test without valgus/varus stress and assessed as pain and/or click at the corresponding joint lines. Using TF joint line tenderness and the McMurray test for the clinical assessment of possible meniscal lesions, current results revealed a significant higher prevalence of positive findings among floor layers compared to graphic designers. However, such positive findings do not necessarily indicate an actual meniscal lesion. Various intra- and periarticular knee disorders may be associated with diverse symptoms and clinical findings, which may mimic meniscal pathology. Meniscal lesions are for example highly correlated with radiographic knee OA among middle-aged and older adults, although interactions between the two pathological conditions are not well understood.[25,26,36,37] Progression of knee OA may increase with concomitant meniscal lesions but knee OA may also increase the progression of degenerative meniscal changes. Still, sensitivity analyses excluding participants with radiographic TF OA from our analyses did not alter the observed difference of positive clinical findings between the two occupational groups. However, knee OA represents a degenerative process evolving over years and radiographic changes constitute later stages of OA. Early stages of knee OA, not radiographic visible yet, may still induce knee symptoms and the possibility of residual confounding must therefore be acknowledged.

An association between kneeling and meniscal lesions has been described in earlier reports by Sharrad and Baker, respectively. [12,13] Kivimäki et al. showed that meniscal lesions that had been verified by a physician, were reported twice as frequently by floor layers compared to painters.[14] However, a subsequent clinical examination of TF joint line tenderness revealed no differences between the two study groups. Negative clinical findings were also found in another Finnish study comparing reinforcement workers with painters.[38] Using painters' as references in these studies could possibly impair a given association as painters' work tasks also include knee straining work positions. Anyhow, these conflicting reports could reflect different aspects of diagnostic tools e.g. self-reported information, physical tests and arthroscopic findings. Knee arthroscopy has been the reference standard for the diagnosis of meniscal lesions in many years. However, arthroscopy is an invasive procedure with certain risks and discomfort for the patients.[39] Opposed to arthroscopy, magnetic resonance imaging (MRI) is a safe and non-invasive method for the evaluation of internal knee pathologies and MRI is commonly accepted as an accurate method for the evaluation of meniscal lesions. [39-42] However, it is still an expensive diagnostic tool and physical tests combined with a case history still remains an important implement in the primary first line diagnosis of possible meniscal lesions.

Studies regarding meniscal kinematics during knee flexion have been analysed in human in vitro models and in MRI in vivo studies. [43-47] Results from these studies have shown that loads transmitted through the menisci increase during knee flexion and that the menisci underwent large posterior displacements on the tibial plateau in deep knee flexion. Higher contact forces and a greater mobility of the menisci during knee flexion could make them more vulnerable to lesions in loaded and prolonged kneeling positions. In addition, getting from kneeling work positions to the standing position many times a day could theoretically predispose to subclinical knee twists and meniscal damage. This could explain current results with a higher prevalence of clinical signs suggesting possible meniscal lesions among floor layers.

Knee pain is a common complaint in the general population. Depending on age, occupational connection and the definition of pain the prevalence has shown to range from 10–60%.[48] The prevalence of knee pain among floor layers in the current study was comparable with results found in other questionnaire surveys dealing with knee pain and occupational knee demands.[2,14,15] This trend remained if the dichotomisation of answer options in the KOOS questionnaire was changed from 0 vs. 1–4 to 0–1 vs. 2–4. Pain during stair walking and difficulty during kneeling and squatting was especially pronounced among floor layers. It was however rather surprisingly that there were only minor differences between the two study groups regarding kneeling and squatting difficulties. This may be due to selection bias as analyses in the age stratum with high participation rates (50–59 years) revealed a significant difference between the two occupational groups. Knee pain during stair walking and catching or locking of the knee joint is two classical symptoms that may indicate possible meniscal pathology. In a study by Abdon et al. among patients undergoing arthroscopy for suspected meniscal lesions the combination of symptoms and signs that indicated the presence of meniscal lesions was studied.[49] They suggested that joint line tenderness in combination with a history of mechanical locking was a better correlate compared to the McMurray test. In the current study we do not have arthroscopic findings but the likelihood of reporting symptoms such as knee pain during stair walking and/or catching of the knee joint was generally high among participants with positive clinical findings suggesting possible meniscal lesions. However, there were also a high proportion of participants with these subjective complaints that did not have clinical signs of possible meniscal lesions. There is therefore a poor correlation between these complaints and clinical findings, and knee pain during stair walking or catching of the knee joint may arise from other knee disorders than meniscal pathology e.g. PF OA.

## Conclusion

Results from the current study indicate that floor layers have a high prevalence of both self-reported and clinical knee morbidity. Clinical knee findings suggesting possible meniscal lesions were significant more prevalent among floor layers compared to graphic designers and an association with occupational kneeling could be possible. However, a causal relation cannot be confirmed due to the cross-sectional study design. To investigate such an association there is a need for future cohort studies (e.g. MRI or arthroscopic) analysing the incidence of occupational related meniscal pathology.

## Competing interests

The authors declare that they have no competing interests.

## Authors' contributions

SR participated in the design of the study, in the acquisition of data, performed the statistical analyses and participated in the interpretation of data. LKJ and JPB participated in the design of the study and in the analyses and the interpretation of data. All authors have been involved in drafting the manuscript and approved the final version of the manuscript.

## Appendix

Knee symptoms and pain during the last week:

**S1**.

Do you have swelling in your knee?

0 (Never), 1 (Rarely), 2 (Sometimes), 3 (Often), 4 (Always)

**S3**.

Does your knee catch or hang up when moving?

0 (Never), 1 (Rarely), 2 (Sometimes), 3 (Often), 4 (Always)

**S6**.

How severe is your knee joint stiffness after first wakening in the morning?

0 (None), 1 (Mild), 2 (Moderate), 3 (Severe), 4 (Extreme)

**P1**.

How often do you experience knee pain?

0 (Never), 1 (Monthly), 2 (Weekly), 3 (Daily), 4 (Always)

**P6**.

Knee pain at night while in bed

0 (None), 1 (Mild), 2 (Moderate), 3 (Severe), 4 (Extreme)

**P7**.

Knee pain going up or down stairs

0 (None), 1 (Mild), 2 (Moderate), 3 (Severe), 4 (Extreme)

Degree of difficulty during the last week due to your knee in the following activities:

**SP1**.

Squatting

0 (None), 1 (Mild), 2 (Moderate), 3 (Severe), 4 (Extreme)

**SP5**.

Kneeling

0 (None), 1 (Mild), 2 (Moderate), 3 (Severe), 4 (Extreme)

## Pre-publication history

The pre-publication history for this paper can be accessed here:


